# Laser-induced layers peeling of sputtering coatings at 1064 nm wavelength

**DOI:** 10.1038/s41598-020-80304-2

**Published:** 2021-02-12

**Authors:** Kesheng Guo, Yanzhi Wang, Ruiyi Chen, Yuhui Zhang, Anna Sytchkova, Meiping Zhu, Kui Yi, Hongbo He, Jianda Shao

**Affiliations:** 1 Laboratory of Thin Film Optics, Key Laboratory of Materials for High Power Laser, Shanghai Institute of Optics and Fine Mechanics, Shanghai, 201800 China; 2 Ji Hua Laboratory, Foshan, 528000 China; 3 CAS Center for Excellence in Ultra-intense Laser Science, Shanghai, China; 4 ENEA Optical Coatings Group, Via Anguillarese 301, Rome, 00123 China

**Keywords:** Optics and photonics, Applied optics

## Abstract

Large-scale layers peeling after the laser irradiation of dual ion beam sputtering coatings is discovered and a model is established to explain it. The laser damage morphologies relate to the laser fluence, showing thermomechanical coupling failure at low energy and coating layers separation at high energy. High-pressure gradients appear in the interaction between laser and coatings, resulting in large-scale layer separation. A two-step laser damage model including defect-induced damage process and ionized air wave damage process is proposed to explain the two phenomena at different energy. At relatively high energies (higher than 20 J/cm^2^), ionization of the air can be initiated, leading to a peeling off effect. The peeling effect is related to the thermomechanical properties of the coating materials.

## Introduction

Optical coatings can increase the transmittance and reflectance of optical elements such as glass and lenses, which are widely used in various laser systems^1–3^. Sputtered coatings have excellent mechanical properties and stability, which are suitable for use in space laser systems^4,5^. Generally, laser-induced damage threshold (LIDT) of dielectric coatings materials is lower than the damage threshold of bulk materials^6,7^. The laser damage problem of dielectric coatings is a key factor in laser systems^8,9^. The laser damage of the sputtered coatings is related to the launch and operation stability of the entire spacecraft mission^10,11^. The research on the mechanism of laser and sputtered coatings is very important.

Nanosecond laser damage is usually attributed to structural defects or absorptive defects^12,13^. Nanoprecursors that initially induce damage are difficult to characterize or observe^14,15^. Analysis of the laser damage morphology can reveal the mechanism behind the damage phenomenon^16–18^. Research on bulk materials such as fused silica found that the multi-longitudinal mode laser interacts with fused silica to form a ripple structure^19–21^, which is related to the laser excited air electrons^22^. Diaz et al. find that the action mechanism of fused silica in vacuum under laser irradiation is related to the ionization of SiO_2_ material on the surface to form a plasma, which also has a ripple structure^23,24^. This phenomenon of laser-excited air or surface matter forming a plasma correlates with the wavelength of the laser^25^. A 1ω frequency laser is more likely to excite air than a 3ω laser^26^. The ripple structure does not appear when the electron beam evaporated coatings interacts with laser, and it mainly manifests as surface ablation^27^. The high temperature of the ionizing wave causes the surface of the evaporated coatings to ablate quickly^28^. The research on the interaction mechanism between the sputtered coatings and laser is rare.

In this work, two different laser damage morphologies of dual ion beam sputtering coatings with high-resolution characterization reveal different damage mechanisms, one of which is large-area film separation. The damage morphologies are related to the laser energy, which show thermomechanical coupling failure at low energy and coating layers separation at high energy. The damage features of sputtering coatings are different from the ring patterns of fused silica or the surface scalding of the e-beam evaporation coatings. High-pressure gradients appear in the interaction between laser and coatings, resulting in large-scale layer separation. Different layer stress parameters make the peeling off effect different. A two-step laser damage model including defect-induced damage process and ionized air wave damage process is proposed to explain the two phenomena.

## Experiments

### Coatings preparation

The fused silica (HPFS 7980, Corning) substrates have no absorption band between 185 and 2500 nm. The index of refraction of substrates is 1.45 and transmittance is above 94% at 1064 nm. The cylindrical substrates have a radius of 25 mm and a thickness of 5 mm. Detailed materials and optical properties of three different coatings (Al_2_O_3_/SiO_2_, Ta_2_O_5_/SiO_2_ and Nb_2_O_5_/SiO_2_) are shown in the Table 1. Before coating, ultrasonic and chemical etching should be used to clean the substrate, mainly to remove surface contamination and polishing deposition contamination of the substrates. Dual ion beam sputtering equipment (Veeco, Ltd.) is used to deposit multilayer coatings.

**Table 1:** Three groups of coatings.

Group name	Coating materials	Optical performance	Layers
I	Al_2_O_3_/SiO_2_	T > 99.5%@1064 nm	11
II	Ta_2_O_5_/SiO_2_	T > 99%@1064 nm	72
III	Nb_2_O_5_/SiO_2_	R > 99%@1064 nm	71

### Laser-induced damage parameters

**Figure 1:**
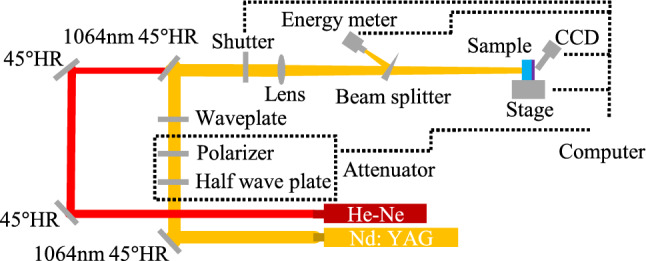
Laser damage test platform.

In the experiment, the 1-on-1 laser damage performance test is carried out according to the standard ISO 21254^29^. The schematic diagram of laser damage test platform is shown in Fig. 1. The incident angle of laser to three samples is 0 degree. Sample I and II are tested on laser exit surface. Sample III is tested on laser incident surface. The pulse width of Nd: YAG laser is 12 ns at 1064 nm (1ω). The facula radius of the incident laser on the coatings is about 200 μm at 1/e^2^ of the maximum intensity. In the laser damage experiment, there are 20 points irradiated by each energy step. The online CCD (charge coupled device) and offline optical microscope can be used to evaluate whether the test area is damaged.

## Experimental results and analysis

### Laser damage probability

**Figure 2:**
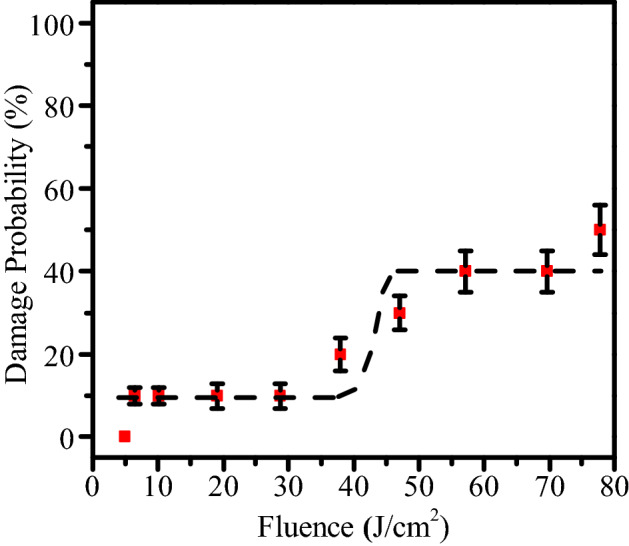
Damage probability curves of Al_2_O_3_/SiO_2_ coatings.

Laser damage probability distribution of the Al_2_O_3_/SiO_2_ coatings is shown in Fig. 2. It can be obtained that within 30 J/cm^2^ energy, the probability of laser damage is low, and is about 40% around 70 J/cm^2^. The two-stage damage probability indicates that there are two different defects. One has a lower density but is prone to laser damage, and the other has a higher density but requires higher energy.

### Laser damage morphology

**Figure 3:**
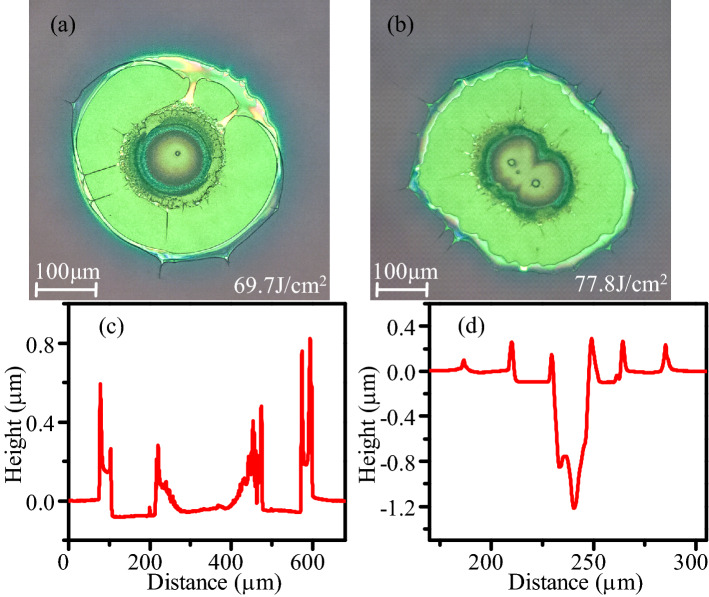
Optical microscope morphologies and damage pits depth of Al_2_O_3_/SiO_2_ coatings after laser damage test, (**c**) and (**d**) correspond to the depth distribution of (**a**) and (**b**), respectively.

Optical microscope (Leica) and optical profiler (Veeco) are used to characterize laser damage morphologies. Morphologies and damage pits depth of Al_2_O_3_/SiO_2_ coatings are shown in Fig. 3(c) and (d) correspond to the depth distribution of (a) and (b), respectively. Figure 3(a) is single defect-induced damage, and (b) is multiple defect-induced damage. Obvious peeling off of coatings layer is observed, and no change in the color of the plasma ablation is observed. From the depth profile of Fig. 3(d), the damage depth is about 1.2 μm, which is close to the substrate. The defects of sample I is possible from interface of coatings and substrate.

**Figure 4:**
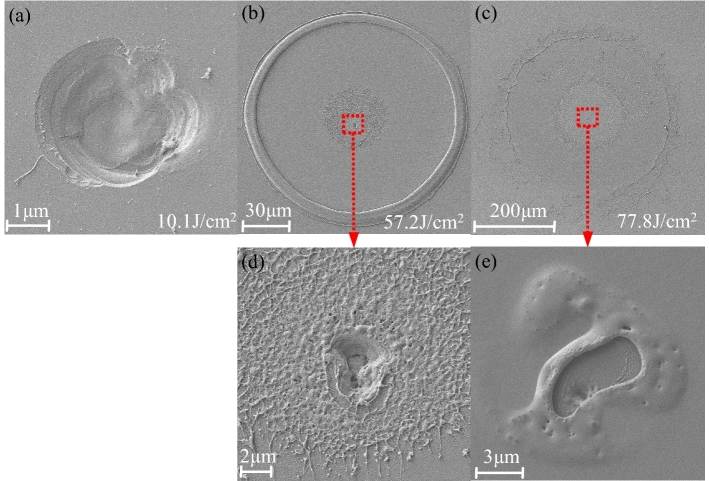
SEM morphologies of Al_2_O_3_/SiO_2_ coatings after laser irradiation: (**a**) close to laser damage threshold; (**b**) middle laser fluence; (**c**) high laser fluence; (**d**) and (**e**) are enlarged images of the center area of (**b**) and (**c**), respectively.

Field emission scanning electron microscopy (FE-SEM; Zeiss) is used to characterize the microscopic morphology of damage pits. Figure 4(a–c) show the damage morphologies of Al_2_O_3_/SiO_2_ coatings at near damage threshold, medium energy, and high energy, respectively. Figure 4(d) and (e) are enlarged views of the central regions of (b) and (c), respectively. Laser damage near the threshold appears as thermal–mechanical coupling failure. The diameter of the damage pit is about 3 μm. The edge contour of the damage pit is clear and shows brittleness distortion, which indicates that the defects are far away from the coatings surface and the thermal effect is not obvious. The central thermal–mechanical coupling damage pit can still be observed at medium energy in Fig. 4(d), but the surrounding coatings are extensively damaged, which is manifested as peeling off. At high energy, the central damage pit is not deeper and only appears as more thermodynamic ablation. The area of ablation and damage of the surrounding peeling layers is larger.

Ta_2_O_5_/SiO_2_ and Nb_2_O_5_/SiO_2_ coatings also show a similar phenomenon, that is, they only show thermal damage at low energy, and at high energy, in addition to thermal damage, the coatings show peeling off effect. The critical energy density of the three coatings is about 20 J/cm^2^.

**Figure 5:**
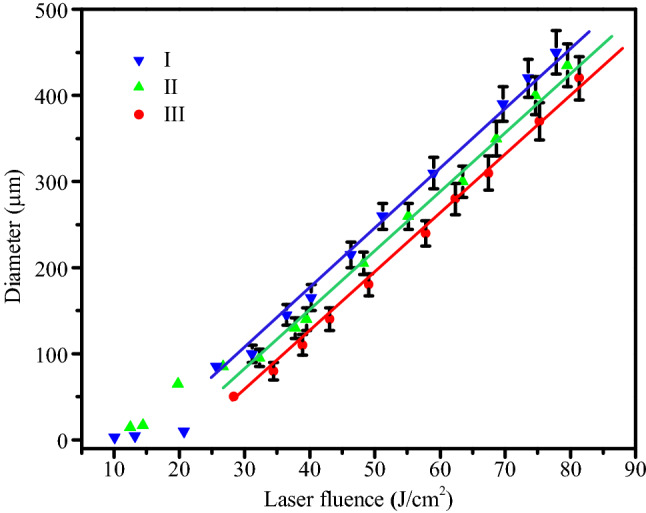
Laser damage pit diameters as function of energy, three different coatings: I-Al_2_O_3_/SiO_2_, II-Ta_2_O_5_/SiO_2_, III-Nb_2_O_5_/SiO_2_.

Figure 5 indicates that the size of the damage pit changes with the laser energy. Some damage pits of sample I and II are observed at relatively low energy, which are relatively small, especially sample I. Sample III is not damaged at relatively low energy, so no damage point was observed at relatively low energy. The damage pit size becomes significantly larger after energy above about 20 J/cm^2^, and with the increase of energy, the development of damage pit size approaches a linear increase. Under the same laser energy, sample I has the largest damage pit size, and sample III has the smallest size. This is related to the thermodynamic properties of the film composition of the samples, which will be explained in detail later.

### Damage mechanism

To explain the correlation between the occurrence of the peeling effect and the laser fluence at 1064 nm. Compared with the shock wave model proposed by Fabbro et al. for the shock wave caused by the laser irradiated material^30^, the shock wave is generated in the solid material, and the propagation speed is the speed of sound level. The propagation speed of shock waves in solid materials is much lower than the surface destruction speed. Therefore, the model we propose is to ionize air to generate plasma, and the speed of expansion and propagation in the air is in the same order of magnitude as the speed of destruction in the experiment. The possible formation of air laser supported detonation waves (LSD) is considered^31^. This happens when the free electron energy *E* can excite the neutral substance in the medium (mainly composed of O_2_ and N_2_ molecules) to ionize^32^. At the beginning of ionization, the maximum energy obtained by the electrons cannot be higher than the following value^33^:

1 $$ E_{m} [{\text{eV}}] \approx 4.9(\lambda [{\mu m}])^{2} I[{\text{GW/cm}}^{{2}} ] $$

Among the Eq. (1), *I* is the laser light intensity, *λ* is the laser wavelength, The energy of air molecules (mainly N_2_ and O_2_) ionized by laser is 12 eV. Equation (1) can be used to calculate the laser power density required for ionizing air as 2.14GW/cm^2^ at 1064 nm. According to the conversion formula ($F = 0.5\sqrt {\pi /\ln 2I\tau }$) of laser energy density and power density, the energy density of the laser can be obtained as 27.34 J/cm^2^. Laser damage will cause the temperature of the coating material around the defect to rise sharply and the absorption will increase^34,35^. The LSD wave front will absorb and reflect the laser^34,36–38^, making the initial electron avalanche ionization energy lower than 27.34 J/cm^2^, which is about 20 J/cm^2^ in our experiment.

Thus, when the energy is low, the laser energy is lower than the ionization energy of the air, and no LSD wave in air can be generated. At this time, the laser and film defects interact with each other, and the defects absorb the laser energy, resulting in thermomechanical coupling damage, such as Fig. 4(a). Due to the deeper defects of the sample II compared to sample I and the strong layer binding force, the thermal effect is more obvious and the damage area is larger in the process of the sample II absorbing the thermal coupling effect of the defect. When the laser energy is greater than the ionization energy of air, LSD waves are generated in the air. Thus, the large-scale emergence of peeling off of coatings is related to a propagation of LSD wave, which is similarly with ring-pattern damage morphologies of the fused silica bulk material^39^. According to the experimental data in Fig. 5, the velocity of propagation of peeling off can be obtained as 21 km/s (laser energy: 70 J/cm^2^, maximum diameter: 500 μm), which is equivalent to the speed of a surface shock wave^23^. Multi-layer coatings deposited by dual ion beam sputtering usually possess high compressive residual stress^40^. The temperature of laser-induced plasma is higher than 10^4^ K, and the pressure is higher than 1 GPa^41^. The laser-induced stress wave propagates horizontally and vertically in the coatings and reflects at the boundary of the coatings, thereby changing the residual stress field of the coatings. At the same time, when the stress wave propagates far away from the center of the laser spot, it attenuates exponentially, and gradually disperses. A stress field distribution similar to the shape of Airy Pattern is formed in the coatings^32^. At the same time, due to the high temperature gradient brought by the LSD wave, the samples are prone to peeling off. Thermodynamic parameters will affect the peeling size of the samples.

The separation of the coating layers originates from the changes in the local stress of different coating layers after the temperature rises, considering the case where the temperature has not reached the melting and vaporization of the coating layers. The change of coatings stress caused by temperature can be explained by the following formula^42^:

2 $$ \sigma_{C} = - \frac{{E_{c} }}{{1 - v_{c} }}\alpha_{c} \Delta T, $$

Among them, *α*_*c*_, *ν*_*c*_, and *E*_*c*_ are the thermal expansion coefficient, Poisson’s ratio and Young modulus of different coating material. Δ*T* is the amount of change in temperature rise. Table 2 shows the mechanical parameters of SiO_2_, Al_2_O_3_, Ta_2_O_5_, and Nb_2_O_5_ coating materials^43,44^. The stress change caused by the same temperature change in the Al_2_O_3_ layer, Ta_2_O_5_, and Nb_2_O_5_ are 63.59 times, 13.53 times, and 8.99 times that of SiO_2_ layer, respectively. This explains that sample I which contains Al_2_O_3_/SiO_2_ layers is more likely to occur peeling effect caused by temperature rise. Thus, the peeling off size of sample I is relatively larger.

**Table 2:** Mechanical parameters of four types of different coating layers.

Materials	*E* (GPa)	*ν*	*α*(1/*K*) × 10^6^
SiO_2_	73	0.17	0.55
Al_2_O_3_	300	0.21	8.1
Ta_2_O_5_	140	0.23	3.6
Nb_2_O_5_	60	0.20	5.8

**Figure 6:**
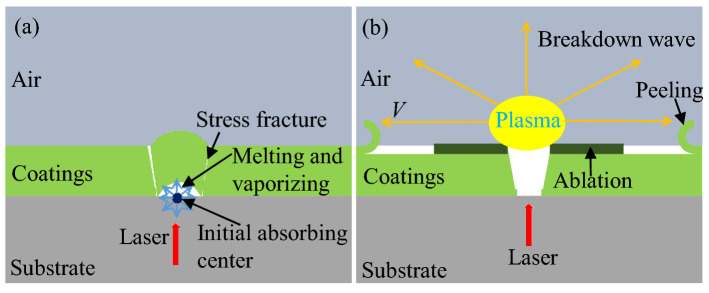
Schematic diagram of laser damage process: (**a**) initial defect absorbing laser energy, thermomechanical coupling effect; (**b**) ionizing air to form plasma, peeling off effects.

Therefore, the laser-induced damage of dual ion beam sputtering coatings is mainly divided into two processes, as shown in the Fig. 6. The first step is the defect absorbing laser energy to induce damage, as can be seen in Fig. 6(a). In nanosecond laser damage realm, the distribution of defects is random, and the laser intensity is Gaussian, so the laser intensity of the defect location is random, and the size of the damage pit is also random. In the defect-induced damage process, damage morphology is also correlated with the thermomechanical parameters of the coatings, the type of defect, number of defects in the spot range, and the distribution depth of defect. In the interaction with the laser, the defect absorbs heat, and the local coatings is melted and gasified, resulting in initial damage to the film. Deeper defects require more layers to be destroyed, and the thermal coupling time is longer and the scale of the laser damage is larger.

The second step is the damage of ionized air waves, as shown in the Fig. 6(b), which only occurs at relatively high energies. Air is a wide band gap dielectric, which is basically transparent to the laser, and basically does not absorb laser energy. In the first step, the broken pieces and residual bonds become the precursor of the ionized air, providing the initial seed electrons. Electron avalanche occurs during laser irradiation, resulting in severe ionization of air and formation of plasma. Plasma almost completely absorbs laser energy. The heated gas expands to form a spherical shock wave in all directions, and the air is heated to dozens of eV. The ionization front expands, forming a temperature gradient, and then the film undergoes stress peeling off.

## Conclusion

The phenomenon of peeling damage of the dual ion beam sputtering coatings was found and explained. A two-step laser damage model is proposed, including defect-induced damage process and ionized air wave damage process. At relatively high energies (higher than 20 J/cm^2^), ionization of the air can be initiated, leading to a peeling off effect. The peeling effect is correlated with the thermomechanical properties of the coatings materials. For coatings with large stress differences, the peeling off effect is more serious. This article is helpful for the analysis of the damage process of the dual ion beam sputtering coatings, which can help improve the ability to resist laser damage from a process and design perspective.
